# Phylogeography of the Assassin Bug *Sphedanolestes impressicollis* in East Asia Inferred From Mitochondrial and Nuclear Gene Sequences

**DOI:** 10.3390/ijms20051234

**Published:** 2019-03-12

**Authors:** Zhenyong Du, Tadashi Ishikawa, Hui Liu, Satoshi Kamitani, Osamu Tadauchi, Wanzhi Cai, Hu Li

**Affiliations:** 1 Department of Entomology and MOA Key Lab of Pest Monitoring and Green Management, College of Plant Protection, China Agricultural University, Beijing 100193, China; caudzy@126.com (Z.D.); liuhui8221@163.com (H.L.); caiwz@cau.edu.cn (W.C.); 2 Laboratory of Entomology, Faculty of Agriculture, Tokyo University of Agriculture, Atsugi, Kanagawa 243-0034, Japan; chuishikawa@gmail.com; 3 Entomological Laboratory, Graduate School of Bioresource and Bioenvironmental Sciences, Kyushu University, Fukuoka 812-8581, Japan; kamitani@agr.kyushu-u.ac.jp (S.K.); tadauchi@agr.kyushu-u.ac.jp (O.T.)

**Keywords:** phylogeography, *Sphedanolestes impressicollis*, population genetic differentiation, land bridge, East Asia

## Abstract

The assassin bug, *Sphedanolestes impressicollis* (Hemiptera: Reduviidae), is widely distributed in East Asia. It is an ideal model for evaluating the effects of climatic fluctuation and geographical events on the distribution patterns of East Asian reduviids. Here, we used two mitochondrial genes and one nuclear gene to investigate the phylogeographic pattern of the assassin bug based on comprehensive sampling in China, Japan, South Korea, Vietnam, and Laos. High levels of genetic differentiation were detected among the geographic populations classified into the northern and southern groups. A significant correlation was detected between genetic and geographical distances. The East China Sea land bridge served as a “dispersal corridor” during Pleistocene glaciation. The estimated divergence time indicated that the northern group may have separated from the eastern Chinese populations when the sea level rapidly rose during the “Ryukyu Coral Sea Stage” and the East China Sea land bridge was completely submerged. Demographic history and ecological niche modeling suggested that appropriate climatic conditions may have accounted for the rapid spread across the Korean Peninsula and Japan during the late Pleistocene. Our study underscores the pivotal roles of the Pleistocene sea level changes and climatic fluctuations in determining the distribution patterns of East Asian reduviids.

## 1. Introduction

During the Quaternary, at least six glacial advances affected the physical and biological environments of the Northern Hemisphere [1]. Glaciation dramatically altered landmass configurations through sea level fluctuation, tectonic uplift and subsidence, sediment deposition, and tidal scouring [2]. The impact of glacial expansions and contractions on heredity largely depends on latitude and topography [3,4]. Phylogeographic studies of various organisms have demonstrated that climatic fluctuations and geographic events played major roles in shaping present-day geographical species distribution, genetic structure, and diversity [5,6,7].

East Asia has long been considered a biodiversity hotspot. Its complicated topography and changeable climatic conditions provided effective refugia for organisms during glaciation. In East Asia, glacial advances were not as extensive as those in Europe or America because of monsoons caused by differential heating between the Asian continent and the Pacific Ocean [8]. The Asian biotic zones were located at higher northern latitudes than those on other continents [9,10]. Climate recovery in East Asia during the interglacial periods differed from that of other continents, primarily because of the ongoing uplift of the Qinghai–Tibetan Plateau during the late Tertiary [11]. Climatic fluctuations during the Quaternary dramatically influenced the evolution and distribution of organisms currently found throughout East Asia [12,13,14]. The eastern edges of this region have undergone drastic changes in paleo-landscape structure due to frequent shallow sea transgressions and regressions between eastern China, the Korean Peninsula, and the Japanese archipelago. These sea level variations have alternately exposed and submerged the East China Sea (ECS) land bridge. Therefore, Eastern China, Southern Japan, and the Korean Peninsula vacillated between separation and combination, which resulted in fragmentation and admixture of the biota population in the Sino-Japanese region [13,15,16]. Nevertheless, the geographic and geological events affecting present-day insect distributions are poorly understood. Most phylogeographic insect studies have been conducted on local regions and have not considered the large spatial scale [17,18,19,20].

The assassin bug *Sphedanolestes impressicollis* Stål belongs to the subfamily Harpactorinae and was first described in 1861 in Hong Kong and thereafter in Japan and South Korea. This assassin bug has been identified on the Japanese islands of Kyushu, Shikoku, and Honshu; throughout South Korea and China; and in parts of Vietnam, Laos, and India [21]. It lives on grasslands and trees in the lowlands and mountainous areas, and feeds on a wide array of insects, such as leaf beetles and lepidopteran larva [22]. *Sphedanolestes impressicollis* is one of the most widely distributed assassin bug species in East Asia and the surrounding areas. It adapts to long-distance diffusion and is relatively sensitive to temperature changes, making it an ideal model for the evaluation of phylogeographic patterns and the assessment of the effects of topography and climatic cycles on the demographic history of East Asian reduviids and other indigenous insects [22]. Since sample collection is difficult, however, very few phylogeographic studies have focused on reduviid species [23,24]. Analyzing the genetic structure of this species helps test hypotheses concerning glacial migration across paleogeographic land bridges and vicariant range segregation caused by inter- and postglacial sea level increases.

**Figure 1:**
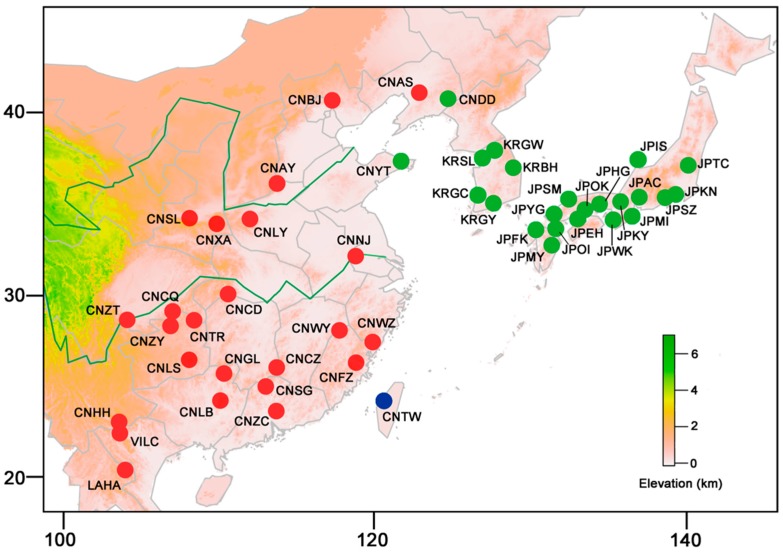
Sample locations of populations used in the present study and spatial population clustering based on the concatenated mitochondrial dataset. Circles in color indicate three clusters classified by BAPS. Population codes and locality details are listed in Table S1.

In this study, the mitochondrial genes cytochrome oxidase subunit I (*COI*) and cytochrome b (*Cytb*) and the nuclear gene elongation factor-1α (*EF-1α*) were used to establish the phylogeographic pattern and correlative demographic history of *Sphedanolestes impressicollis* based on comprehensive sampling from various geographic distributions (Figure 1). We detected high levels of genetic differentiation among these populations. We classified them into the northern group (N group) consisting of all individuals from Japan and Korean Peninsula and Dandong (CNDD) and Yantai (CNYT) in Mainland China (Table S1). The southern group (S group) included individuals from most populations of Mainland China, Taiwan Island, and Vietnam-Laos. There were significant correlations between the genetic and geographical distances among adjacent populations. Divergence time estimation indicated that the N group radiated from the eastern Chinese populations when the sea level quickly rose during the “Ryukyu Coral Sea Stage” and entirely submerged the heretofore exposed ECS land bridge. Inferred demographic history and ecological niche modeling (ENM) demonstrated that climatic conditions were conducive to the rapid dispersal of *Sphedanolestes impressicollis* across the Korean Peninsula and Japan during the late Pleistocene. Our results supported the hypothesis that the ECS land bridge was a “dispersal corridor” for East Asian insects [16,25]. It also underscores the pivotal roles of the Pleistocene sea level changes and climatic fluctuations in shaping the distribution patterns of East Asian reduviids.

## 2. Results

### 2.1. Genetic Diversity

In the concatenated mitochondrial dataset (1764 bp), 225 sites were variable among these individuals. There were 131 parsimony informative sites and 94 singleton variable sites. These polymorphic sites defined 152 haplotypes across the species ranges. The overall haplotype (Hd) and the nucleotide (π) diversities for all populations were 0.9905 and 0.00744, respectively. The average haplotype diversity range for all populations from China, Japan, South Korea, Vietnam, and Laos was 0.000–1.000 and the mean nucleotide diversity range was 0.000–0.0085 (Table S1). Of the large populations (N > 5), Hd = 0.756–1.000 and π = 0.00113–0.0085. Chinese populations presented with the greatest mitochondrial diversity, followed by Vietnam-Laos, South Korea, and Japan. The S group exhibited an obviously higher level of genetic diversity than the N group (Table 1).

For the *EF-1a* sequences (898 bp) obtained from 87 individuals, eight haplotypes were defined by seven segregating sites. The overall Hd and π for all populations were 0.713 and 0.00164, respectively (Table S2). Only two different haplotypes were defined by two segregating sites in the Japanese populations, four in the Chinese populations, and two in the Vietnam-Laos population. No segregating site was found in the Korean populations. The average Hd range for all populations of China, Japan, and Vietnam-Laos was 0.499–0.833 and the average π range was 0.00092–0.00130 (Table S2). For the phylogenetic groups defined by the mitochondrial dataset, the numbers of segregating sites and haplotypes of the S group were greater than those of the N group, even though the former had a smaller sample size (Table S2). In view of the very limited sample size and DNA polymorphism of the *EF-1α* gene sequences, this dataset was mainly used as a reference in the present study.

### 2.2. Population Genetic Structure

**Figure 2:**
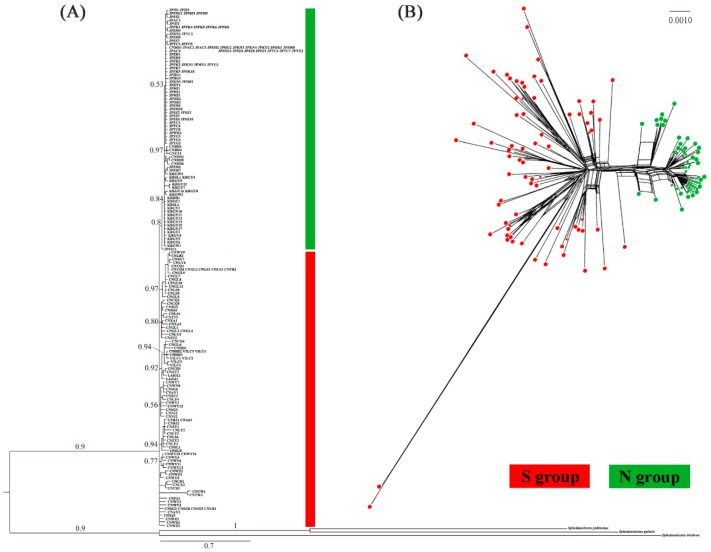
(**A**) Bayesian tree based on concatenated mitochondrial haplotypes. Bayesian posterior probabilities are shown at each node. Population codes are listed in Table S1. (**B**) Split network based on the concatenated mitochondrial dataset.

BAPS analysis classified all populations into three groups with distinct population structures. The first cluster consisted of the two Chinese populations CNDD and CNYT and all Japanese and South Korean populations. Other Chinese populations and those of Vietnam and Laos were included in the second cluster. The third cluster comprised the population of Taiwan Island (Figure 1). BI and ML analyses of 152 haplotypes detected from the concatenated mitochondrial dataset identified concordant topologies (Figure 2A and Figure S1). Only one major monophyletic haplotype group was revealed, including the haplotypes of individuals from Japan (JP), South Korea (KR), CNDD, and CNYT, and these were designated as the N group. The other haplotypes were defined as the S group, which presented with a broad geographical distribution and weak phylogenetic structure and ranged from China to Vietnam and Laos. The split network analysis based on the mitochondrial haplotypes generated results consistent with those of the BI/ML trees, which also identified the N and S groups (Figure 2B). The S group included haplotypes from most populations of Mainland China, Taiwan Island, Vietnam, and Laos.

Analysis of molecular variance (AMOVA) revealed significant genetic variance based on both the concatenated mitochondrial dataset and the nuclear gene at different hierarchical levels (Table 2). For the concatenated mitochondrial dataset, AMOVA based on two groups indicated a significant level of differentiation among groups (44.70% of the variation; *p* < 0.0001). In contrast, it was 18.77% among populations within groups and 36.53% within populations. Pairwise *F_ST_* based on the concatenated mitochondrial dataset between the N and S groups was 0.46026 (*p* < 0.0001). For the nuclear *EF-1a* gene sequence, AMOVA disclosed that 51.14% of the variation was between N and S (*p* < 0.0001). Only 27.42% of the variation was among populations within groups and 21.44% was within populations (Table 2). The data indicated significant genetic variance when populations were classified into two groups by phylogenetic tree and basic and specific (BASP) analysis. Significant correlations between the genetic and geographical distances among populations were detected for all samples, including both N and S groups (*p* < 0.05; Figure S2).

### 2.3. Haplotype Distribution

**Figure 3:**
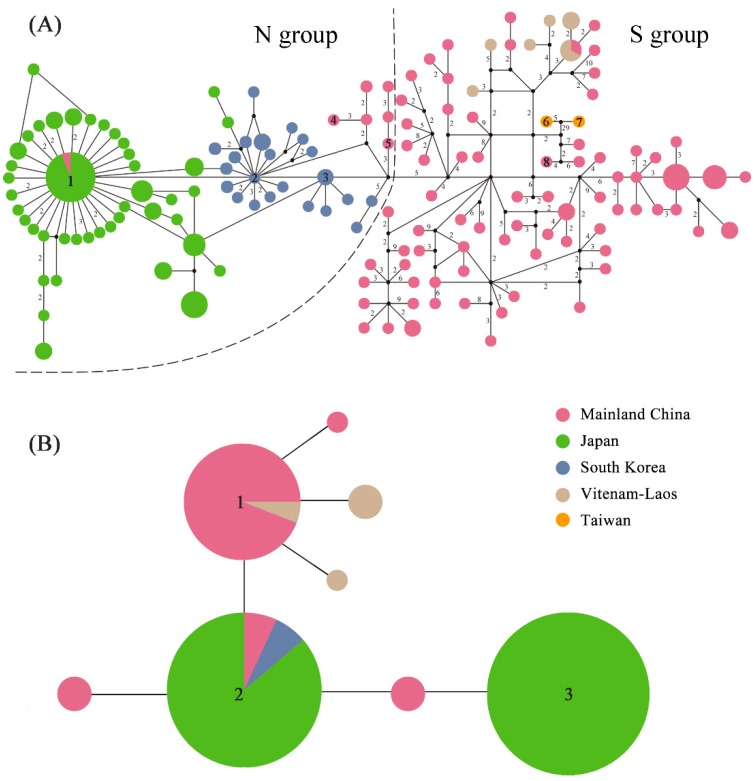
(**A**) Haplotype network estimated from the concatenated mitochondrial dataset and (**B**) nuclear gene sequences. Small black circles represent missing haplotypes. Distances between linked haplotypes corresponded to one mutation, except where otherwise specified. Circle sizes are proportional to numbers of haplotypes. Numbers in the circles represent haplotype numbers.

Median-joining network results based on 152 mitochondrial haplotypes showed a structure resembling that resolved by phylogenetic analyses (Figure 2A and Figure S1). Both the N and S groups formed a subnetwork separated by > 5 mutational steps without a shared haplotype (Figure 3A). The N group had a star-like topology. Haplotype 1 had a widespread interior geographic distribution from Japan. Interior haplotype 2 was distributed in South Korea. In contrast, the S group displayed a complex topology dominated by haplotypes from populations in Mainland China, Taiwan Island, and Vietnam-Laos (Figure 3A). The star-like haplotypes indicated a typical expansion process. Among the 152 haplotypes, 20 occurred in > 2 individuals, whereas the others occurred in only one individual. The most frequently occurring haplotype 1 was found in 17 individuals from Japan and one CNDD individual (Figure 3A). Haplotypes 2 and 3 and their surrounding haplotypes were localized to the South Korean populations. The single CNYT haplotype (haplotype 4), haplotype 5, and another four surrounding haplotypes from CNDD were clustered as the limited Chinese populations of the N group (Figure 3A). Network analysis indicated that the haplotypes of CNDD, CNYT, and South Korea connected the eastern Chinese haplotypes (CNWZ, etc.) in the S group and the Japanese haplotypes in the N group. Haplotypes 6 and 7 from Taiwan Island were classified as a single group in BASP (Figure 1) and found inside the S group. They were genetically closer to Haplotype 8 and three others from Nanjing (CNNJ) in eastern China. Nevertheless, their mutations were greater than those of the others in the S group.

For the nuclear *EF-1a* gene sequence, only eight haplotypes were identified for all 87 sequences (Figure 3B). Three highly shared haplotypes dominated the haplotype network. Haplotype 1 was found in multiple populations of the S group. This finding resembled those based on the mitochondrial dataset (Figure 3A). Haplotype 2 was geographically widespread; it covered CNLY and CNDD in China and all of the Japanese and South Korean populations. Haplotype 3 was only common among the Japanese populations.

### 2.4. Estimated Divergence Time

The Bayesian evolutionary analysis sampling tree (BEAST) and estimated divergence time for pivotal nodes are shown in Figure S3. The divergence time between the N and S groups was 0.91 Ma (95% HPD: 0.33–1.78 Ma). The most recent common ancestor (MRCA) of the N group was dated to 0.51 Ma (0.20–1.04 Ma) and that for the S group was 0.83 Ma (0.32–1.66 Ma). The MRCA of the Taiwanese population was dated to 0.13 Ma (0.03–0.32 Ma).

### 2.5. Demographic History

**Figure 4:**
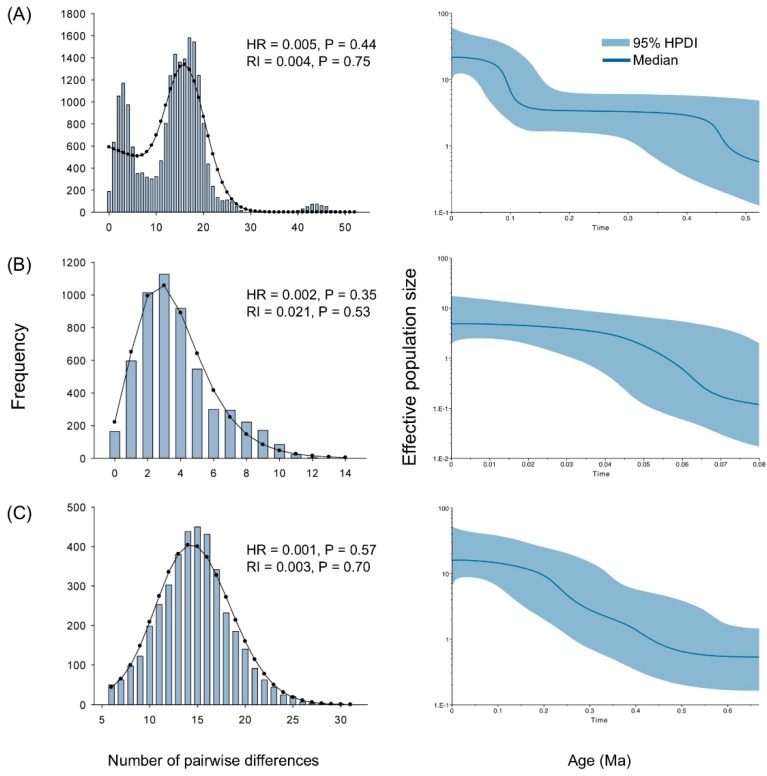
Mismatch distributions (left) and Bayesian skyline plots (right). (**A**) All samples, (**B**) N group, and (**C**) S group were calculated based on the concatenated mitochondrial dataset. Observed mismatch distribution is denoted by vertical bars. Expected distribution under population expansion model is represented by red lines. HR and RI indices are shown. For Bayesian skyline plots, median estimated effective population sizes (middle lines) are enclosed within 95% highest posterior density intervals (shaded areas).

Both Tajima’s D and Fu’s Fs results were negative and statistically significant for all samples and the N and S groups (Table 2). This observation supported the hypothesis that the populations underwent marked expansion. The mismatch distributions detected in all samples and both groups were unimodal and characteristic of populations which have spread on a very large scale (Figure 4). The age expansion parameter *τ* localizes the mismatch distribution crest and roughly estimates the time at which rapid population expansion began for *Sphedanolestes impressicollis*. *τ* was 16.90000 units of mutational time in all samples (Table 1). *τ* = 2μt = 16.90000 was used to estimate the expansion time for all samples and t = τ/2μ = 16.9000/ (2 × 1.15 × 10^−8^ × 1764) = 416,543 generations (416,543 y). *τ* for the S group (14.97266) was much larger than that of the N group (2.38867). The calculated expansion time for the N group was 58,875 generations (58,875 y) and that for the S group was 369,039 generations (369,039 y). BSP analysis revealed two significant increases in the effective population size for all samples which occurred ~0.1 Ma and ~0.5 Ma (Figure 4). For the N and S groups, continuous expansion started from ~0.08 Ma and ~0.5 Ma, respectively.

### 2.6. Ecological Niche Modeling

**Figure 5:**
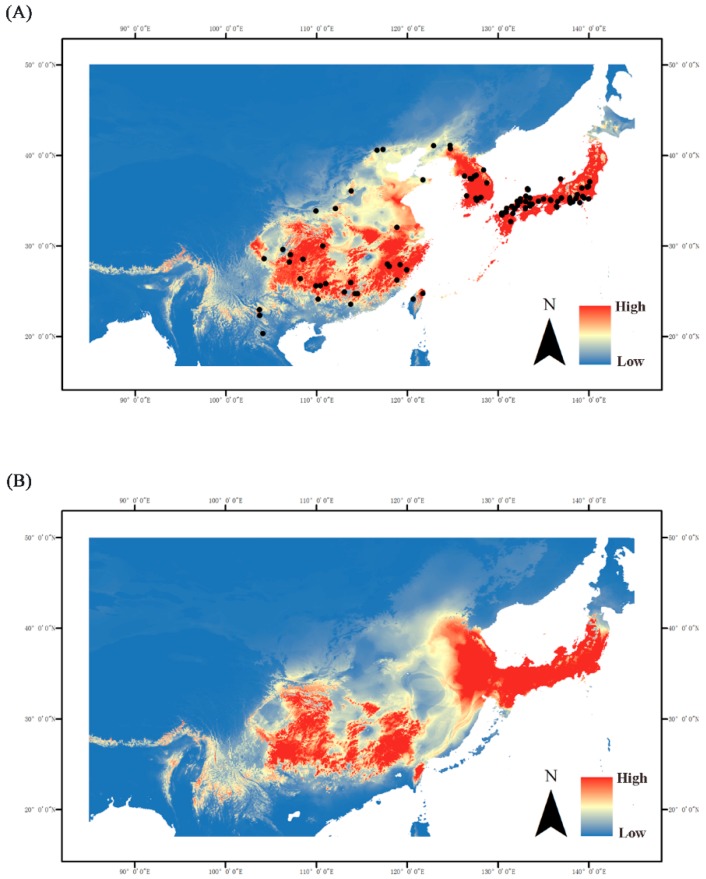
Hindcasting ecological niche models in East Asia during the (**A**) current and (**B**) LGM period. Black dots represent recording localities used to fit the ecological niche model. Blue indicates low suitability, while red indicates high suitability.

AUC of 0.9562 and 0.9621 were calculated by ENM analysis, which indicated a good performance in the model predictions. The current model prediction aligned well with the actual sample distribution (Figure 5A). Both the N and S groups occupied highly suitable areas on a large scale. The predictive current distributions of the S group had larger suitable areas than those for the Last Glacial Maximum (LGM) prediction. Therefore, the S group widened its range after glaciation. In the LGM prediction, the most suitable areas were mainly located in southern China and did not substantially differ from the current situation. The highly suitable areas at present already cover Korean Peninsula and Japan in addition to southern China (Figure 5B).

## 3. Discussion

### 3.1. Sphedanolestes Impressicollis Distribution Pattern in East Asia

Our analyses revealed that all *Sphedanolestes impressicollis* populations in East Asia can be classified into two main lineages, namely, the N and S groups. The N group covers the entire sampling region of Japan and South Korea and two populations in northern China, while the S group is restricted to China, Vietnam, and Laos (Figure 1, Figure 2, Figure 3 and Figure S1). In the present study, the highest haplotype and nucleotide diversity levels were detected in the Chinese populations followed by Vietnam-Laos, South Korea, and Japan. A large degree of genetic diversity may reflect long evolutionary histories and limited interpopulation gene flow [26,27]. It is confirmed that *Sphedanolestes impressicollis* is native to China. The observed low levels of genetic variation in the South Korean and Japanese populations are attributed to recurring bottlenecks, founder events, and genetic drift [28,29]. The pairwise *F_ST_* results revealed a general pattern. Much higher *F_ST_* values were found between the Chinese and Japanese populations and between the Chinese and South Korean populations than those between populations within the same countries (Table S3). These strong interpopulation differences may be explained by geographical barriers which separated populations in different regions and blocked gene flow. The East China and Yellow Seas may have imposed significant geographical barriers to gene flow among Chinese populations and among Japanese/South Korean populations. Genetic differentiation was higher between the Japanese and South Korean populations than within the Japanese populations (Table S3). Old straits were most likely the main obstacles in the determination of genetic boundaries among island populations [30]. The Korea Strait was a substantial barrier to gene flow between these two regions. The observed haplotype distribution strongly supported the dispersal pattern from eastern China to Japan. The South Korean populations connected the Japanese and eastern Chinese haplotypes. However, we localized the CNYT and CNDD of China to the N group. Therefore, individuals of the N group may have recently returned to China from Korean Peninsula and Japan via natural dispersion at the northeastern Chinese boundary and marine trade in the ECS and Yellow Sea [23], respectively. Consequently, the N and S groups have coexisted in northern China and reduced it to a narrow secondary contact zone.

The Vietnam-Laos population was the most closely genetically related to the populations from southwestern China, including the CNCQ, CNZT, CNHH, and CNGL haplotypes (Figure 1, Figure 2 and Figure 3A). Based on the *EF-1α* gene, a common haplotype was shared by these populations (Figure 4B). This finding resembled those obtained from the mitochondrial dataset (Figure 3A). In view of the low level of genetic diversity, the Vietnam and Laos populations may have originated in southwestern China. The haplotype distribution of the Taiwan Island population (Figure 3A) indicated that it was closely genetically related to the populations from southeastern China (CNNJ and CNWZ, etc.). These results corroborate the theory that the Taiwanese population originated in southeastern China. For the Taiwan Island population, however, the significantly larger mutation steps and the BAPS division suggested long-term isolation rather than a recent invasion event. Combining these data with the estimated MRCA time, we inferred that the Taiwan Island population was isolated from the southeastern Chinese population because the land bridge was submerged during the Pleistocene glaciation and separated Taiwan Island from Mainland China. The Taiwan Strait was also a barrier to gene flow between the Taiwanese and original Mainland Chinese populations.

### 3.2. Historical Demography in the Sino-Japanese Region

From the Miocene to the Pleistocene, the ECS land bridge formed three times between the Eurasian continent and Japanese archipelago at ~5.0–7.0 Ma, ~1.3–2.0 Ma, and ~0.015–0.2 Ma [31]. The estimated divergence time of the two main lineages (0.91 Ma) and the MRCA of the N (0.51 Ma) and S (0.83 Ma) groups coincided with the “Ryukyu Coral Sea Stage” (0.2–1.3 Ma) [32] when the sea level rapidly rose and completely submerged the heretofore exposed ECS land bridge. Before this event, the assassin bug had already spread into the Korean Peninsula and Japanese archipelago via the ECS land bridge. Our data substantiate the possibility that the ECS land bridge may have served as a “dispersal corridor” for East Asian insects and connected Mainland China, the Korean Peninsula, and Southern Japan [33,34,35]. This hypothesis has also been supported by the results of studies on hemipterans [16,25]. Since their climates were unfavorable, however, South Korea and Japan may have restricted their populations to relatively narrow distributions. Increases in sea level may have caused population extinctions on the ECS land bridge and long-term isolation between the Korean-Japanese and native Chinese populations. Demographic inference, mismatch distributions, and neutral tests strongly indicate that *Sphedanolestes impressicollis* significantly expanded at ~0.5 Ma and again at ~0.1 Ma (Table S1; Figure 4). Continuous expansion of the S group occurred during the middle Pleistocene (0.126–0.781 Ma) [36], whereas the N group dispersed during the late Pleistocene. The first expansion during the middle Pleistocene may have been the rapid emergence of the native Chinese population from their glacier refugia [9]. In the second expansion, the narrowly distributed Korean and Southern Japanese populations quickly spread, possibly because of increasing climatic conformity. Haplotype distribution analysis (Figure 4) disclosed that the expansion process was sequential and the South Korean populations served as a transition between the eastern Chinese and Japanese populations. The ENM analysis of the LGM period (Figure 4) showed that the Korean Peninsula and Japanese archipelago were already highly suitable for *Sphedanolestes impressicollis* and did not markedly differ from the current condition in these areas. Climatic fluctuation in the late Pleistocene enabled *Sphedanolestes impressicollis* to often disperse across the Korean Peninsula and Japan and fill the ecological niches.

### 3.3. Phylogeography of Native Chinese Populations

The impact of the Pleistocene glaciation cycles may have dramatically influenced population divergence patterns in many organisms [3,4]. Populations were isolated in different refugial areas during the glaciation periods and extended their ranges by colonization as favorable climatic and ecological conditions resumed. The suitable areas at present predicted by ENM were larger than those at LGM. Therefore, a range expansion may have occurred in the S group after glaciation. Glacial refugia may have harbored most of the intraspecific diversity [37]. In the present study, comparatively higher nucleotide and haplotype diversities were found in populations from southwestern China (CNCQ, CNLB, etc.). Moreover, numerous unique haplotypes were identified in these two populations. The southwestern plateau in China may have undergone three to five glaciations between the late and middle Pleistocene (0.78–1.0 Ma) [38]. At lower elevations in this area, environmental heterogeneity persisted [10]. This region is considered a biodiversity hotspot and has retained many relic plant species from the Tertiary. It was also a refugium for temperate and subtropical species during the cold phases of the Quaternary [39]. Relatively high nucleotide and haplotype diversity (Table S1) were discovered in the Chongqing population (CNCQ) of the Sichuan Basin in China. Both the current and LGM period ENM predictions demonstrated high suitability levels in the Sichuan Basin. Therefore, it might have been a refugium for *Sphedanolestes impressicollis*.

In the present study, phylogenetic haplotype trees based on a concatenated mitochondrial dataset disclosed generally unresolved topologies and comparatively few substantial indications of the intraspecific phylogeographical structure in China (Figure 2A and Figure S1). The demographic expansion of *Sphedanolestes impressicollis* may explain the frequent gene flow and lack of geographic association among mitochondrial haplotypes. Growth of the Chinese populations may have occurred during ~0.3–0.5 Ma, which was consistent with the onset of the Penultimate Interglacial Period of China (~0.33–0.46 Ma) [40,41]. The subsequent environmental changes were moderate during the climate oscillations in southeastern China. Populations grew steadily from their refugia throughout the LGM. Warm and wet summer monsoons in East Asia and eastern China since the mid-late Pleistocene [9] further expanded the available habitat for *Sphedanolestes impressicollis*. Recent studies showed a similar demographic pattern of certain organisms in China, whose population expansions started before LGM [11,15,42,43]. Continuous expansion maintains frequent gene flow among different populations and prevents the formation of a strong phylogeographic structure. Genetic structure may be conserved in certain fragmented habitats of the wide *Sphedanolestes impressicollis* distributions across East Asia. Since sample size and molecular markers were limited, however, we could not elaborate on the potential genetic structure or accurately localize the refugia in the present study. A higher level of individual sampling and more genome-wide molecular markers are needed to elucidate the detailed evolutionary history of *Sphedanolestes impressicollis*.

## 4. Material and Methods

### 4.1. Sampling, DNA Extraction, Amplification, and Sequencing

In total, 199 *Sphedanolestes impressicollis* individuals were sampled from 48 localities in Mainland China, Taiwan Island, South Korea, Japan, Vietnam, and Laos (Table S1, Figure 1). They were stored at −20 °C in absolute ethanol until DNA extraction. Total genomic DNA was extracted from the legs of each insect and analyzed with a DNeasy blood and tissue kit, according to the manufacturer’s protocols (QIAGEN, Hilden, Germany). Amplification primers for the two mitochondrial genes (*COI* and *Cytb*) and the nuclear gene (*EF-1α*) are listed in Table S4 [44,45,46]. PCR was performed with TaKaRa ExTaq^TM^ (TaKaRa Biomedical, Kusatsu, Shiga, Japan) under the following conditions: initial denaturation at 94 °C for 5 min; 35 cycles at 94 °C for 30 s; 45–50 °C for 30 s; 72 °C for 1.5 min; and a final single extension at 72 °C for 6 min. All PCR products were purified and sequenced in both directions with an ABI 3730XL DNA analyzer (Applied Biosystems, Foster City, CA, USA). All sequences were assembled in Geneious v. 11.1.3 (http://www.geneious.com/) and deposited into GenBank under the following accession numbers: (*COI*: MK346629–MK346827; *Cytb*: MK346343–MK346541; *EF-1α*: MK346542–MK346628).

### 4.2. Phylogenetic Analysis

Each gene was independently aligned with Mafft v. 7 [47] and the G-INS-i strategy. Clustering of individuals was performed for the concatenated mitochondrial dataset with BAPS v. 6.0 [48] based on the spatial clustering of groups of individuals model. Twenty runs (K = 20) were made to ensure consistency and convergence of the results. Phylogenetic analyses were performed by the Bayesian inference (BI) and maximum likelihood (ML) methods and implemented in MrBayes v. 3.1.1 [49] and RAxML v. 8.2 [50]. According to the Akaike Information Criterion (AIC), the GTR + I + G model was selected with JModelTest v. 0.1.1 [51]. For the Bayesian analysis, two simultaneous runs of one million generations were conducted for the dataset. Trees were sampled every 1000 generations and the first 25% was discarded as burn-in. ML analysis was performed with RAxML and the node support values were assessed by 1000 bootstrap replicates. *Sphedanolestes trichrous*, *Sphedanolestes gularis,* and *Sphedanolestes pubinotus* were designated as outgroups for the phylogenetic analyses. Split networks based on the concatenated mitochondrial dataset were constructed with Splitstee v. 4.13.1 [52], which created a valuable framework for analyses by trees and networks. To detect a significant reticulation signature, the neighbor-net method was used for construction under a K2P distance model. Other network constructions were created by the median-joining method [53] with Network v. 4.5.0.0 (www.fluxus-engineering.com) based on the concatenated mitochondrial dataset and the nuclear gene sequences.

### 4.3. Genetic Diversity and Population Differentiation

To test genetic diversity and differentiation, the number of segregating sites (S), number of haplotypes (H), haplotype diversity (Hd), and nucleotide diversity (π) were calculated for all populations with DnaSP v. 5.0 [54]. Hierarchical analysis of molecular variance (AMOVA) among populations and between defined groups was implemented with 1000 permutations in Arlequin v. 3.5 [55]. Pairwise *F_ST_* values were estimated between large populations (N > 5). Mantel tests were run based on the mitochondrial dataset to identify the isolation-by-distance model for all samples and the N and S groups. Correlations between genetic distances (*F_ST_*) and linear geographic distance (ln Km) were plotted with 999 replicates in the ade4 v. 1.7 module of R.

### 4.4. Divergence Time Estimation

BEAST v. 2.5.1 [56] was used to estimate the divergence time based on the concatenated mitochondrial dataset. No fossils were available to calibrate the nodes. Therefore, a controversial substitution rate of 1.15%/Ma was proposed [57] and the best fit GTR + I + G model was adopted in the present study. An uncorrelated lognormal relaxed clock model was measured by ucld.stdev and coefficients of variation near one were used. Two independent MCMC runs per 300 million generations were made with tree sampling every 1000 generations. Tracer v. 1.7 was used to verify whether the MCMC runs reached a stationary distribution based on the effective sample sizes (ESS) of each estimated parameter. ESS > 200 for the posterior, prior, and tree likelihoods. TreeAnnotator v. 1.5.3 was used to calculate the consensus tree and annotate the mean height of divergence times using 25% trees as burn-in.

### 4.5. Demographic History

Tajima’s D [58] and Fu’s Fs [59] were used to assess the demographic history of *Sphedanolestes impressicollis*. To confirm whether expansion occurred, mismatch distributions were examined. These are typically ragged or multimodal for populations at stationary demographic equilibrium. In contrast, they are smooth or unimodal for populations which have undergone demographic expansion [60]. The fit of the observed data to the expected data was compared by the sum of squares deviations (SSD) and Harpending’s raggedness index (r) estimated from 1,000 parametric bootstrap replicates in Arlequin. The expansion time was estimated in generations (*t*) and calculated from *t* = *τ*/2*μ*, where *τ* (tau) is the expansion time in mutation units and *μ* is the mutation rate per generation for the DNA sequence. The *Sphedanolestes impressicollis* generation time was taken to be 1 y. To identify the changes in effective population over time, Bayesian skyline plots (BSP) were generated in BEAST v. 2.5.1 [56], based on the concatenated mitochondrial dataset. The same models and substitution rates were used as those for divergence time estimation. The coalescent Bayesian skyline model was applied for tree priors. Output plots were visualized in Tracer, with the first 10% discarded as burn-in.

### 4.6. Ecological Niche Modeling

A total of 104 localities were used for the ENM, including 48 sample localities and another 56 records of dry preserved specimens (Table S5). Modern climate data was obtained from a geographic information system (GIS) in the form of ESRI (Environmental Systems Research Institute, Redlands, CA, USA) grids. To model the effect of Pleistocene climatic oscillations on distributions, paleoclimate ecological niche models were constructed under the climatic conditions during the LGM 18–21 ka [61]. The Interdisciplinary Research on Climate Model (MIROC) was used in the LGM prediction. The current and LGM climate layers were generated in ArcGIS v. 10.0 (http://www.esri.com/software/arcgis). The bioclimatic variables most likely to affect the distribution were mean temperature (BIO1), mean diurnal temperature range (BIO2), maximum temperature of the warmest month (BIO5), minimum temperature of the coldest month (BIO6), annual mean precipitation (BIO12), precipitation of the wettest month (BIO13), and precipitation of the driest month (BIO14). They were obtained from the Worldclim database at a 2.5-min resolution (http://www.worldclim.org/). ENM analysis was executed with maximum entropy in Maxent v. 3.3.3k [62]. The analysis was run in ten replicates under default program conditions. Eighty percent of the recorded localities were used to train and 20% were used to test the model. Model performance was evaluated according to the areas under the curve (AUC) of the receiver operating a characteristic (ROC) plot. The index of suitability ranged from 0–1 and the output of the predicted distributions on the grid maps was visualized in ArcGIS.

## 5. Conclusions

In this study, we resolved the phylogeographic pattern among *Sphedanolestes impressicollis* populations in East Asia, which were classified into the N and S groups. A significant correlation was detected between genetic and geographical distances. The ECS land bridge served as a “dispersal corridor” during Pleistocene glaciation. The N group separated from the eastern Chinese populations when the sea level rapidly rose during the “Ryukyu Coral Sea Stage” and the ECS land bridge was completely submerged. The rapid spread across the Korean Peninsula and Japan during the late Pleistocene benefited from appropriate climatic conditions for *Sphedanolestes impressicollis.* Our study highlights the crucial roles of the Pleistocene sea level changes and climatic fluctuations that determined the distribution patterns of East Asian reduviids.

## Figures and Tables

**Table 1:** Genetic diversity, neutrality tests, and statistics of mismatch distribution based on the concatenated mitochondrial dataset.

Group	N	Nh	S	π	Tajima’s D	Fu’s Fs	τ
All samples	199	152	225	0.00744	−2.087 *	−23.945 *	16.90000
N group	105	71	83	0.00215	−2.476 *	−25.989 *	2.38867
S group	94	81	189	0.00865	−1.972 *	−24.186 *	14.97266

N, sample size; Nh, number of haplotypes; S, number of segregating sites; π, nucleotide diversity; * *p* < 0.001; *τ*, the observed value of the age expansion parameter.

**Table 2:** Hierarchical analysis of molecular variance (AMOVA) based on mitochondrial and nuclear datasets.

Dataset	Source of variation	d.f.	SSD	Percentage	Fixation Indices
Mitochondrial dataset	Among groups	1	392.480	44.70	Φ_CT_ = 0.44698 *
	Among populations within groups	46	436.791	18.77	Φ_SC_ = 0.33949 *
	Within populations	151	469.413	36.53	Φ_ST =_ 0.63473 *
Nuclear gene	Among groups	1	21.423	51.14	Φ_CT_ = 0.56121 *
	Among populations within groups	22	27.434	27.42	Φ_SC_ = 0.51144 *
	Within populations	63	14.361	21.44	Φ_ST_ = 0.78562 *

d.f. degree of freedom, SSD Sum of squares, * *p* < 0.0001.

## Supplementary Materials

Supplementary materials can be found at https://www.mdpi.com/1422-0067/20/5/1234/s1.
