# Supplementary material for: Phylogeography of the Assassin Bug Sphedanolestes impressicollis in East Asia Inferred From Mitochondrial and Nuclear Gene Sequences
Source: Int J Mol Sci. 2019 Mar 12;20(5):1234. doi: 10.3390/ijms20051234 (PMC6429140; doi:10.3390/ijms20051234)
Supplement: Supplementary file 1 [file ijms-20-01234-s001.zip › supplementary_materials_3.8/Table S2.docx]

**Table S3** Genetic diversity based on the EF-1α sequences for *Sphedanolestes impressicollis.*

| Defined population | N | S | Nh | Hd | *π* |
| --- | --- | --- | --- | --- | --- |
| China | 23 | 4 | 5 | 0.514 | 0.00092 |
| Vietnam-Laos | 4 | 2 | 3 | 0.833 | 0.00130 |
| South Korea | 2 | 0 | 1 | 0.000 | 0.00000 |
| Japan | 58 | 2 | 2 | 0.499 | 0.00111 |
| N group | 61 | 2 | 2 | 0.505 | 0.00112 |
| S group | 26 | 6 | 7 | 0.572 | 0.00102 |
| All | 87 | 7 | 8 | 0.713 | 0.00164 |

N Number of sequences, S number of segregating sites, Nh number of haplotypes, Hd haplotype diversity, π nucleotide diversity
