# Supplementary material for: Phylogeography of the Assassin Bug Sphedanolestes impressicollis in East Asia Inferred From Mitochondrial and Nuclear Gene Sequences
Source: Int J Mol Sci. 2019 Mar 12;20(5):1234. doi: 10.3390/ijms20051234 (PMC6429140; doi:10.3390/ijms20051234)
Supplement: Supplementary file 1 [file ijms-20-01234-s001.zip › supplementary_materials_3.8/Table S3.docx]

| **Table S4** Pairwise *F_ST_* values calculated based on mitochondrial dataset. | | | | | | | | | | | | | | | | |
| --- | --- | --- | --- | --- | --- | --- | --- | --- | --- | --- | --- | --- | --- | --- | --- | --- |
| Population | CNCQ | CNWY | CNSG | CNGL | CNLS | CNLY | CNDD | CNWZ | JPFK | JPKN | JPSM | JPSZ | JPTC | JPYG | KRGY | VILC |
| CNCQ | 0 | 0.07554 | 0.0249 | 0.00492^N^ | 0.08624^N^ | 0.16 | 0.43087 | 0.06745^N^ | 0.6 | 0.5212 | 0.62186 | 0.55776 | 0.56901 | 0.51496 | 0.55797 | 0.3166 |
| CNWY | 0.07554 | 0 | 0.11983 | 0.18388 | 0.31491 | 0.16809 | 0.40215 | 0.12523 | 0.53134 | 0.47032 | 0.54251 | 0.48726 | 0.50872 | 0.47132 | 0.48974 | 0.39548 |
| CNSG | 0.0249^N^ | 0.11983 | 0 | 0.04552^N^ | 0.16827 | 0.19117 | 0.46454 | 0.11941 | 0.59912 | 0.52734 | 0.5949 | 0.54109 | 0.55482 | 0.51815 | 0.52798 | 0.46833 |
| CNGL | 0.00492^N^ | 0.18388 | 0.04552^N^ | 0 | 0.00329^N^ | 0.29418 | 0.49469 | 0.15012 | 0.62214 | 0.56173 | 0.62648 | 0.58214 | 0.59174 | 0.55936 | 0.57852 | 0.43639 |
| CNLS | 0.08624^N^ | 0.31491 | 0.16827 | 0.00329^N^ | 0 | 0.41882 | 0.63551 | 0.26076 | 0.7661 | 0.73525 | 0.77058 | 0.72572 | 0.74101 | 0.72437 | 0.69983 | 0.62136 |
| CNLY | 0.16 | 0.16809 | 0.19117 | 0.29418 | 0.41882 | 0 | 0.50309 | 0.2064 | 0.64427 | 0.58881 | 0.6515 | 0.60478 | 0.62128 | 0.57457 | 0.5807 | 0.50309 |
| CNDD | 0.43087 | 0.40215 | 0.46454 | 0.49469 | 0.63551 | 0.50309 | 0 | 0.40856 | 0.48401 | 0.37301 | 0.41679 | 0.32383 | 0.39393 | 0.37335 | 0.25105 | 0.71532 |
| CNWZ | 0.06745^N^ | 0.12523 | 0.11941 | 0.15012 | 0.26076 | 0.2064 | 0.40856 | 0 | 0.59665 | 0.50397 | 0.59979 | 0.52425 | 0.55504 | 0.48568 | 0.52438 | 0.4645 |
| JPFK | 0.6 | 0.53134 | 0.59912 | 0.62214 | 0.7661 | 0.64427 | 0.48401 | 0.59665 | 0 | 0.32425 | 0.36079 | 0.3182 | 0.33003 | 0.30854 | 0.3457 | 0.82192 |
| JPKN | 0.5212 | 0.47032 | 0.52734 | 0.56173 | 0.73525 | 0.58881 | 0.37301 | 0.50397 | 0.32425 | 0 | -0.00222^N^ | -0.02605^N^ | -0.04287^N^ | -0.05263^N^ | 0.17039 | 0.81452 |
| JPSM | 0.62186 | 0.54251 | 0.5949 | 0.62648 | 0.77058 | 0.6515 | 0.41679 | 0.59979 | 0.36079 | -0.00222^N^ | 0 | 0.06526^N^ | 0.07182 | 0.01936^N^ | 0.19449 | 0.82423 |
| JPSZ | 0.55776 | 0.48726 | 0.54109 | 0.58214 | 0.72572 | 0.60478 | 0.32383 | 0.52425 | 0.3182 | -0.02605^N^ | 0.06526^N^ | 0 | 0.06201^N^ | 0.017^N^ | 0.17142 | 0.78296 |
| JPTC | 0.56901 | 0.50872 | 0.55482 | 0.59174 | 0.74101 | 0.62128 | 0.39393 | 0.55504 | 0.33003 | -0.04287^N^ | 0.07182 | 0.06201^N^ | 0 | -0.00143^N^ | 0.20475 | 0.80911 |
| JPYG | 0.51496 | 0.47132 | 0.51815 | 0.55936 | 0.72437 | 0.57457 | 0.37335 | 0.48568 | 0.30854 | -0.05263^N^ | 0.01936^N^ | 0.017^N^ | -0.00143^N^ | 0 | 0.1749 | 0.80153 |
| KRGY | 0.55797 | 0.48974 | 0.52798 | 0.57852 | 0.69983 | 0.5807 | 0.25105 | 0.52438 | 0.3457 | 0.17039 | 0.19449 | 0.17142 | 0.20475 | 0.1749 | 0 | 0.74524 |
| VILC | 0.3166 | 0.39548 | 0.46833 | 0.43639 | 0.62136 | 0.50309 | 0.71532 | 0.4645 | 0.82192 | 0.81452 | 0.82423 | 0.78296 | 0.80911 | 0.80153 | 0.74524 | 0 |

^N^, P > 0.05
