# Supplementary material for: Phylogeography of the Assassin Bug Sphedanolestes impressicollis in East Asia Inferred From Mitochondrial and Nuclear Gene Sequences
Source: Int J Mol Sci. 2019 Mar 12;20(5):1234. doi: 10.3390/ijms20051234 (PMC6429140; doi:10.3390/ijms20051234)
Supplement: Supplementary file 1 [file ijms-20-01234-s001.zip › supplementary_materials_3.8/Table S4.docx]

**Table S2** Primers for amplification and sequencing.

| Gene | Primer name | Primer sequence 5'-3' | Reference |
| --- | --- | --- | --- |
| *COI* | Cl-J-1718  Cl-N-2191 | GGAGGATTTGGAAATTGATTAGTTCC  CCCGGTAAAATTAAAATATAAACTTC | [43] |
|  | C1-J-2183  TL2-N-3014 | CAACATTTATTTTGATTTTTTGG  TCCAATGCACTAATCTGCCATATTA |  |
| *Cytb* | 7432F  7433R | GGACGWGGWATTTATTATGGATC  GC(AT)CCAATTCA(AG)GTTA(AG)TAA | [44] |
| *EF-1α* | F  R | GGACACAGAGATTTCATCAARAA  TTGCAAAGCTTCRTGRTGCATTT | [45] |
