# Supplementary material for: Phylogeography of the Assassin Bug Sphedanolestes impressicollis in East Asia Inferred From Mitochondrial and Nuclear Gene Sequences
Source: Int J Mol Sci. 2019 Mar 12;20(5):1234. doi: 10.3390/ijms20051234 (PMC6429140; doi:10.3390/ijms20051234)
Supplement: Supplementary file 1 [file ijms-20-01234-s001.zip › supplementary_materials_3.8/Table S5.docx]

**Table S5** Distributions information used in the ENM analysis.

| Species | localities | Longitude (E) | Latitude (N) |
| --- | --- | --- | --- |
| *Sphedanolestes impressicollis* | 1 | 117.3355 | 40.6439 |
| *Sphedanolestes impressicollis* | 2 | 116.6814 | 40.5546 |
| *Sphedanolestes impressicollis* | 3 | 107.1279 | 29.0495 |
| *Sphedanolestes impressicollis* | 4 | 106.2848 | 29.6110 |
| *Sphedanolestes impressicollis* | 5 | 118.8635 | 26.2212 |
| *Sphedanolestes impressicollis* | 6 | 118.0358 | 27.7568 |
| *Sphedanolestes impressicollis* | 7 | 113.0828 | 24.9133 |
| *Sphedanolestes impressicollis* | 8 | 114.2609 | 24.7251 |
| *Sphedanolestes impressicollis* | 9 | 113.7721 | 23.5687 |
| *Sphedanolestes impressicollis* | 10 | 111.0144 | 25.8240 |
| *Sphedanolestes impressicollis* | 11 | 109.9352 | 25.5983 |
| *Sphedanolestes impressicollis* | 12 | 110.1885 | 24.1332 |
| *Sphedanolestes impressicollis* | 13 | 110.4293 | 25.6194 |
| *Sphedanolestes impressicollis* | 14 | 108.2008 | 26.3766 |
| *Sphedanolestes impressicollis* | 15 | 107.0133 | 28.2412 |
| *Sphedanolestes impressicollis* | 16 | 108.4944 | 28.5441 |
| *Sphedanolestes impressicollis* | 17 | 112.0959 | 34.1347 |
| *Sphedanolestes impressicollis* | 18 | 113.8191 | 36.0831 |
| *Sphedanolestes impressicollis* | 19 | 112.0857 | 34.1345 |
| *Sphedanolestes impressicollis* | 20 | 110.6804 | 30.0053 |
| *Sphedanolestes impressicollis* | 21 | 113.7843 | 25.9630 |
| *Sphedanolestes impressicollis* | 22 | 118.8479 | 32.0709 |
| *Sphedanolestes impressicollis* | 23 | 114.5301 | 24.7424 |
| *Sphedanolestes impressicollis* | 24 | 117.8170 | 28.0014 |
| *Sphedanolestes impressicollis* | 25 | 118.0355 | 27.7567 |
| *Sphedanolestes impressicollis* | 26 | 122.9032 | 41.0670 |
| *Sphedanolestes impressicollis* | 27 | 124.7521 | 40.7305 |
| *Sphedanolestes impressicollis* | 28 | 124.7540 | 41.1893 |
| *Sphedanolestes impressicollis* | 29 | 124.7240 | 41.0893 |
| *Sphedanolestes impressicollis* | 30 | 121.7410 | 37.3024 |
| *Sphedanolestes impressicollis* | 31 | 109.9410 | 33.8628 |
| *Sphedanolestes impressicollis* | 32 | 121.7480 | 24.7511 |
| *Sphedanolestes impressicollis* | 33 | 120.6585 | 24.1279 |
| *Sphedanolestes impressicollis* | 34 | 103.7014 | 22.9651 |
| *Sphedanolestes impressicollis* | 35 | 104.2341 | 28.5782 |
| *Sphedanolestes impressicollis* | 36 | 119.2096 | 27.9336 |
| *Sphedanolestes impressicollis* | 37 | 119.9353 | 27.3508 |
| *Sphedanolestes impressicollis* | 38 | 136.5897 | 35.6117 |
| *Sphedanolestes impressicollis* | 39 | 139.9935 | 35.1966 |
| *Sphedanolestes impressicollis* | 40 | 133.0334 | 34.1200 |
| *Sphedanolestes impressicollis* | 41 | 133.0135 | 34.2297 |
| *Sphedanolestes impressicollis* | 42 | 130.3751 | 33.5189 |
| *Sphedanolestes impressicollis* | 43 | 130.9094 | 33.7808 |
| *Sphedanolestes impressicollis* | 44 | 130.8032 | 33.7432 |
| *Sphedanolestes impressicollis* | 45 | 130.4250 | 33.6267 |
| *Sphedanolestes impressicollis* | 46 | 139.2384 | 36.3984 |
| *Sphedanolestes impressicollis* | 47 | 134.4438 | 34.9347 |
| *Sphedanolestes impressicollis* | 48 | 135.0352 | 35.1760 |
| *Sphedanolestes impressicollis* | 49 | 133.0574 | 34.8952 |
| *Sphedanolestes impressicollis* | 50 | 132.1769 | 34.4819 |
| *Sphedanolestes impressicollis* | 51 | 136.9004 | 37.3890 |
| *Sphedanolestes impressicollis* | 52 | 136.9897 | 35.3117 |
| *Sphedanolestes impressicollis* | 53 | 139.3230 | 35.4764 |
| *Sphedanolestes impressicollis* | 54 | 135.7864 | 35.0863 |
| *Sphedanolestes impressicollis* | 55 | 136.5032 | 34.2787 |
| *Sphedanolestes impressicollis* | 56 | 136.4257 | 34.3672 |
| *Sphedanolestes impressicollis* | 57 | 136.5925 | 34.9080 |
| *Sphedanolestes impressicollis* | 58 | 131.3892 | 32.6778 |
| *Sphedanolestes impressicollis* | 59 | 137.9411 | 35.3227 |
| *Sphedanolestes impressicollis* | 60 | 131.6472 | 33.5933 |
| *Sphedanolestes impressicollis* | 61 | 133.8999 | 34.8420 |
| *Sphedanolestes impressicollis* | 62 | 133.7555 | 34.6005 |
| *Sphedanolestes impressicollis* | 63 | 133.5372 | 34.3852 |
| *Sphedanolestes impressicollis* | 64 | 130.4462 | 33.4169 |
| *Sphedanolestes impressicollis* | 65 | 135.8312 | 35.0305 |
| *Sphedanolestes impressicollis* | 66 | 132.4836 | 35.2105 |
| *Sphedanolestes impressicollis* | 67 | 139.4809 | 35.2999 |
| *Sphedanolestes impressicollis* | 68 | 133.3307 | 36.2613 |
| *Sphedanolestes impressicollis* | 69 | 132.2713 | 34.9719 |
| *Sphedanolestes impressicollis* | 70 | 132.1179 | 34.9513 |
| *Sphedanolestes impressicollis* | 71 | 133.0796 | 35.5201 |
| *Sphedanolestes impressicollis* | 72 | 132.4395 | 34.8958 |
| *Sphedanolestes impressicollis* | 73 | 133.3222 | 36.2096 |
| *Sphedanolestes impressicollis* | 74 | 133.2504 | 36.3243 |
| *Sphedanolestes impressicollis* | 75 | 138.6218 | 35.2665 |
| *Sphedanolestes impressicollis* | 76 | 138.5786 | 35.2250 |
| *Sphedanolestes impressicollis* | 77 | 138.0030 | 34.7701 |
| *Sphedanolestes impressicollis* | 78 | 137.8850 | 35.0598 |
| *Sphedanolestes impressicollis* | 79 | 138.2531 | 34.8705 |
| *Sphedanolestes impressicollis* | 80 | 139.0357 | 34.7744 |
| *Sphedanolestes impressicollis* | 81 | 140.1011 | 37.0983 |
| *Sphedanolestes impressicollis* | 82 | 139.9853 | 36.5949 |
| *Sphedanolestes impressicollis* | 83 | 133.5217 | 35.2928 |
| *Sphedanolestes impressicollis* | 84 | 133.4999 | 34.6420 |
| *Sphedanolestes impressicollis* | 85 | 131.5274 | 34.3802 |
| *Sphedanolestes impressicollis* | 86 | 130.9585 | 34.1607 |
| *Sphedanolestes impressicollis* | 87 | 131.8146 | 34.0620 |
| *Sphedanolestes impressicollis* | 88 | 131.8609 | 34.1461 |
| *Sphedanolestes impressicollis* | 89 | 138.0367 | 35.6747 |
| *Sphedanolestes impressicollis* | 90 | 127.0946 | 37.3897 |
| *Sphedanolestes impressicollis* | 91 | 128.7692 | 36.9701 |
| *Sphedanolestes impressicollis* | 92 | 127.6157 | 37.8066 |
| *Sphedanolestes impressicollis* | 93 | 126.3233 | 37.7069 |
| *Sphedanolestes impressicollis* | 94 | 127.3795 | 37.6843 |
| *Sphedanolestes impressicollis* | 95 | 126.5522 | 35.5245 |
| *Sphedanolestes impressicollis* | 96 | 127.5688 | 35.2807 |
| *Sphedanolestes impressicollis* | 97 | 126.9519 | 37.4599 |
| *Sphedanolestes impressicollis* | 98 | 127.9618 | 35.5223 |
| *Sphedanolestes impressicollis* | 99 | 128.1126 | 35.3575 |
| *Sphedanolestes impressicollis* | 100 | 127.6618 | 35.0130 |
| *Sphedanolestes impressicollis* | 101 | 128.4031 | 38.3704 |
| *Sphedanolestes impressicollis* | 102 | 104.1561 | 20.3467 |
| *Sphedanolestes impressicollis* | 103 | 104.1001 | 20.3254 |
| *Sphedanolestes impressicollis* | 104 | 103.7500 | 22.3333 |
