# Supplementary material for: Phylogeography of the Assassin Bug Sphedanolestes impressicollis in East Asia Inferred From Mitochondrial and Nuclear Gene Sequences
Source: Int J Mol Sci. 2019 Mar 12;20(5):1234. doi: 10.3390/ijms20051234 (PMC6429140; doi:10.3390/ijms20051234)
Supplement: Supplementary file 1 [file ijms-20-01234-s001.zip › supplementary_materials_3.8/Table S1.docx]

| **Table S1** Sample localities and nucleotide polymorphisms of *Sphedanolestes impressicollis.* | | | | | |
| --- | --- | --- | --- | --- | --- |
| Population | Sample locality | Code | Latitude | Longitude | N |
| **China** | | | | | **93** |
| 1 | Dandong City, Liaoning Province, China | CNDD | 40.7305 | 124.7521 | 6 |
| 2 | Anshan City, Liaoning Province, China | CNAS | 41.0670 | 122.9032 | 1 |
| 3 | Miyun District, Beijing City, China | CNBJ | 40.6439 | 117.3355 | 2 |
| 4 | Yantai City, Shandong Province, China | CNYT | 37.3024 | 121.7410 | 1 |
| 5 | Xi'an City, Shaanxi Province, China | CNXA | 34.1634 | 108.2221 | 2 |
| 6 | Shangluo City, Shaanxi Province, China | CNSL | 33.8628 | 109.9410 | 1 |
| 7 | Anyang City, Henan Province, China | CNAY | 36.0831 | 113.8191 | 2 |
| 8 | Luoyang City, Henan Province, China | CNLY | 34.1345 | 112.0857 | 6 |
| 9 | Changde City, Hunan Province, China | CNCD | 30.0053 | 110.6804 | 1 |
| 10 | Chenzhou City, Hunan Province, China | CNCZ | 25.9630 | 113.7843 | 2 |
| 11 | Nanjing City, Jiangsu Province, China | CNNJ | 32.0709 | 118.8479 | 3 |
| 12 | Wenzhou City, Zhejiang Province, China | CNWZ | 27.3508 | 119.9353 | 5 |
| 13 | Wuyishan City, Fujian Province, China | CNWY | 28.0014 | 117.8170 | 14 |
| 14 | Fuzhou City, Fujian Province, China | CNFZ | 26.2212 | 118.8635 | 1 |
| 15 | Shaoguan City, Guangdong Province, China | CNSG | 24.9133 | 113.0828 | 9 |
| 16 | Zengcheng City, Guangdong Province, China | CNZC | 23.5687 | 113.7721 | 1 |
| 17 | Guilin City, Guangxi Province, China | CNGL | 25.6194 | 110.4293 | 11 |
| 18 | Laibin City, Guangxi Province Province, China | CNLB | 24.1332 | 110.1885 | 2 |
| 19 | Zunyi City, Guizhou Province, China | CNZY | 28.2412 | 107.0133 | 2 |
| 20 | Tongren City, Guizhou Province, China | CNTR | 28.5441 | 108.4944 | 1 |
| 21 | Leishan County, Guizhou Province, China | CNLS | 26.3766 | 108.2008 | 6 |
| 22 | Zhaotong City, Yunnan Province, China | CNZT | 28.5782 | 104.2341 | 3 |
| 23 | Honghe City, Yunnan Province, China | CNHH | 22.9651 | 103.7014 | 3 |
| 24 | Nanchuan District, Chongqing City, China | CNCQ | 29.0495 | 107.1279 | 6 |
| 25 | Taichung County,Taiwan Province, China | CNTW | 24.1279 | 120.6585 | 2 |
| **Vietnam-Laos** | | | | | **8** |
| 26 | Hoang Lien son, Vietnam | VILC | 22.3333 | 103.7500 | 6 |
| 27 | Houaphan Province, Laos | LAHA | 20.3254 | 104.1001 | 2 |
| **South Korea** |  |  |  |  | **24** |
| 28 | Chun-cheon-si, Gang-won-do, Korea | KRGW | 37.8891 | 127.7396 | 3 |
| 29 | Gwan-ak-gu, Seoul, Korea | KRSL | 37.4676 | 126.9434 | 2 |
| 30 | Bong-hwa-gun, Gyeong-sang-buk-do, Korea | KRBH | 36.9351 | 128.9230 | 1 |
| 31 | Go-chang-gun, Jeol-la-buk-do, Korea | KRGC | 35.4389 | 126.6753 | 1 |
| 32 | Gwang-yang-si, Jeollanam-do, Korea | KRGY | 34.9868 | 127.6519 | 17 |
| **Japan** | | | | | **74** |
| 33 | Nishiusuki-gun, Miyazaki Prefecture, Japan | JPMY | 32.6778 | 131.3892 | 1 |
| 34 | Kunisaki-shi, Oita Prefecture, Japan | JPOI | 33.5933 | 131.6472 | 1 |
| 35 | Fukuoka-shi, Fukuoka Prefecture, Japan | JPFK | 33.5189 | 130.3751 | 10 |
| 36 | Imabari-shi, Ehime Prefecture, Japan | JPEH | 34.1200 | 133.0334 | 3 |
| 37 | Hagi-shi, Yamaguchi Prefecture, Japan | JPYG | 34.3802 | 131.5274 | 5 |
| 38 | Toriichôtorii, Ôda-shi , Shimane Prefecture, Japan | JPSM | 35.2105 | 132.4836 | 12 |
| 39 | Ibara-shi, Okayama Prefecture, Japan | JPOK | 34.6420 | 133.4999 | 3 |
| 40 | Akô-gun, Hyôgo Prefecture, Japan | JPHG | 34.9347 | 134.4438 | 3 |
| 41 | Kyoto-shi, Kyoto Prefecture, Japan | JPKY | 35.0863 | 135.7864 | 2 |
| 42 | Arita-gun, Wakayama Prefecture, Japan | JPWK | 34.0715 | 135.3283 | 1 |
| 43 | Minamiise-chô, Mie Prefecture, Japan | JPMI | 34.2787 | 136.5032 | 3 |
| 44 | Komaki-shi, Aichi Prefecture, Japan | JPAC | 35.3117 | 136.9897 | 4 |
| 45 | Fujinomiya-shi, Shizuoka Prefecture, Japan | JPSZ | 35.2891 | 138.6491 | 11 |
| 46 | Atsugi-shi, Kanagawa Prefecture, Japan | JPKN | 35.4842 | 139.3213 | 5 |
| 47 | Wajima-shi, Ishikawa Prefecture, Japan | JPIS | 37.3891 | 136.8993 | 2 |
| 48 | Nasu-gun, Tochigi Prefecture, Japan | JPTC | 37.0805 | 140.1218 | 8 |
| **All** | | | | | **199** |
| N sample size, S number of segregating sites, Nh number of haplotypes, Hd haplotype diversity, π nucleotide diversity | | | | | |
