# Supplementary figures and images for: Phylogeography of the Assassin Bug Sphedanolestes impressicollis in East Asia Inferred From Mitochondrial and Nuclear Gene Sequences
Source: Int J Mol Sci. 2019 Mar 12;20(5):1234. doi: 10.3390/ijms20051234 (PMC6429140; doi:10.3390/ijms20051234)

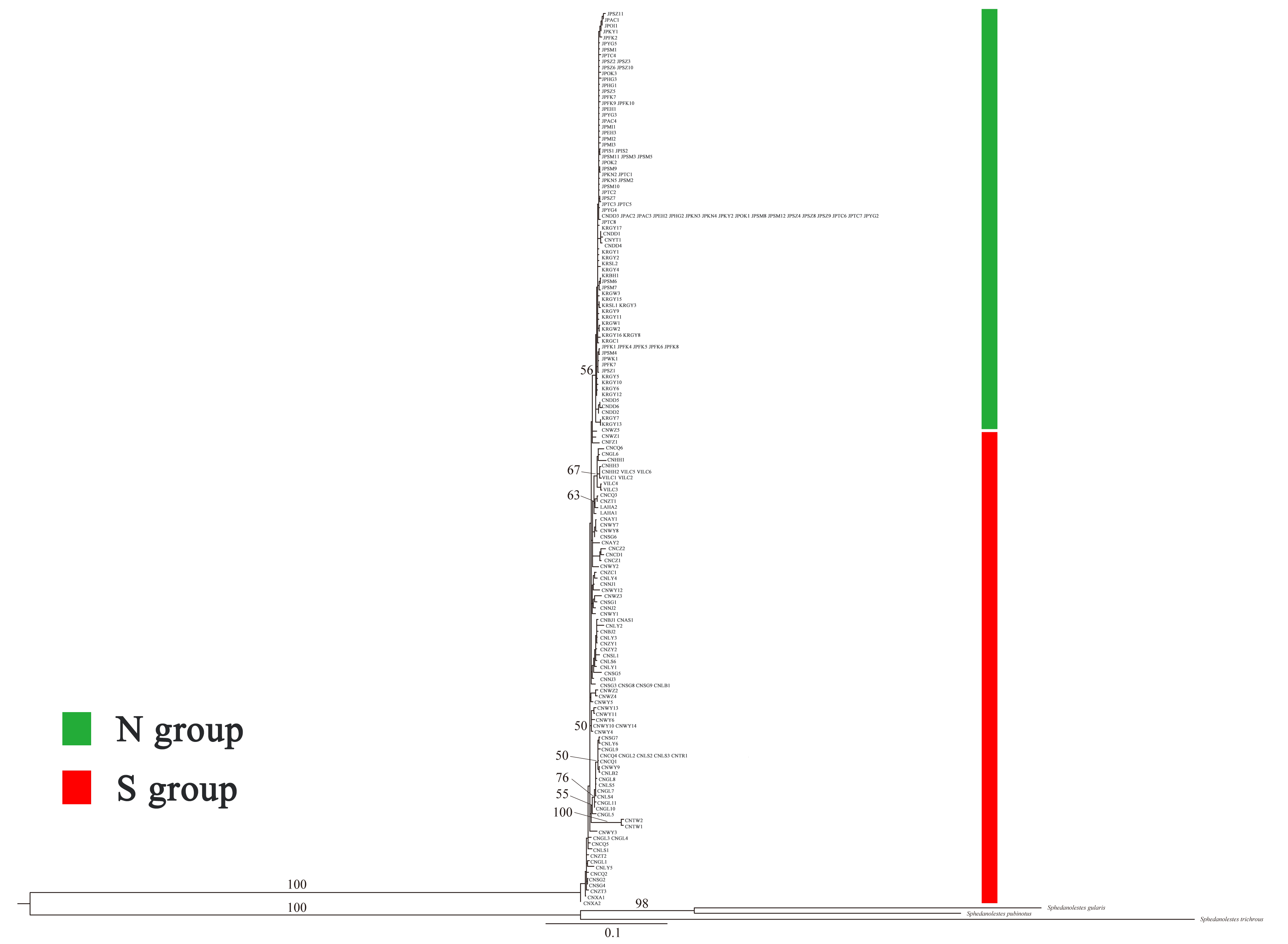

Supplement: Supplementary file 1 [file ijms-20-01234-s001.zip › supplementary_materials_3.8/Fig. S1.tif]

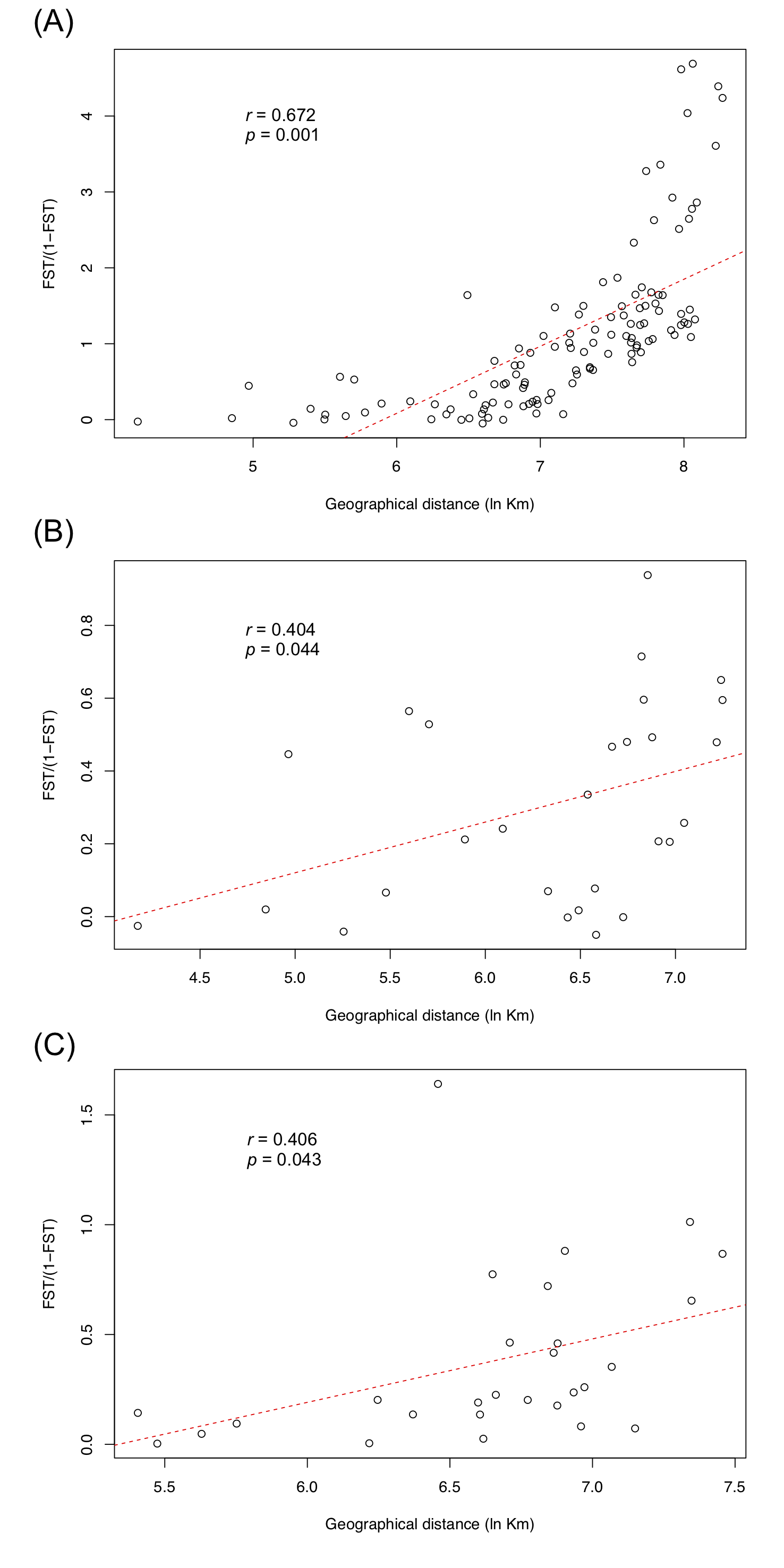

Supplement: Supplementary file 1 [file ijms-20-01234-s001.zip › supplementary_materials_3.8/Fig. S2.tif]

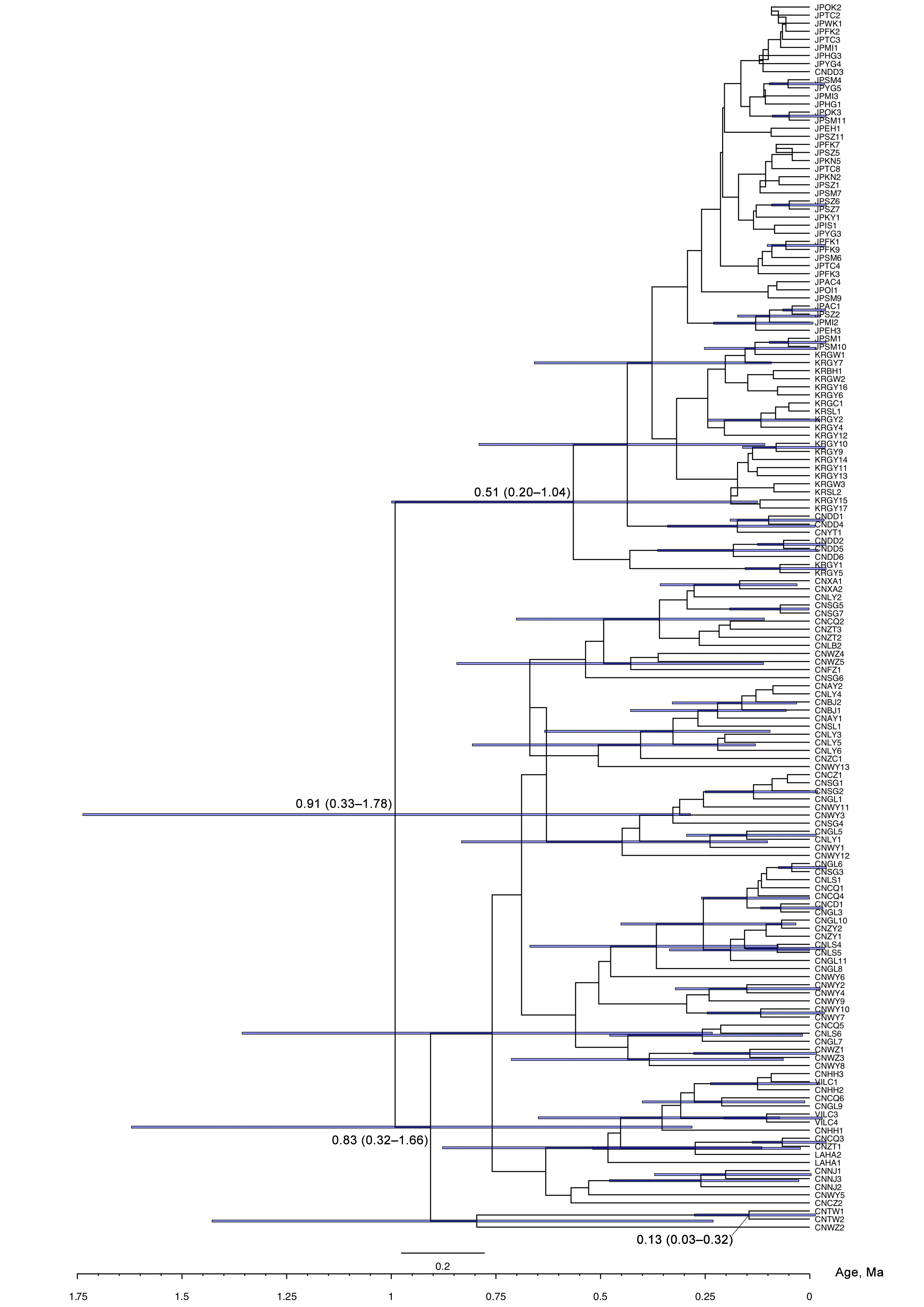

Supplement: Supplementary file 1 [file ijms-20-01234-s001.zip › supplementary_materials_3.8/Fig. S3.tif]
